# *Schistosoma* and *Strongyloides* screening in migrants initiating HIV Care in Canada: a cross sectional study

**DOI:** 10.1186/s12879-020-4779-4

**Published:** 2020-01-28

**Authors:** Jessica McLellan, M. John Gill, Stephen Vaughan, Bonnie Meatherall

**Affiliations:** 1 Department of Medicine, The University of Calgary, Calgary, Alberta Canada; 2 Division of Infectious Disease, The University of Calgary, Calgary, Alberta Canada

**Keywords:** HIV, *Schistosoma*, *Strongyloides*, Seroprevalence, Immigrant, Screening, Eosinophilia

## Abstract

**Background:**

Following migration from *Schistosoma* and *Strongyloides* endemic to non-endemic regions, people remain at high risk for adverse sequelae from these chronic infections. HIV co-infected persons are particularly vulnerable to the serious and potentially fatal consequences of untreated helminth infection. While general screening guidelines exist for parasitic infection screening in immigrant populations, they remain silent on HIV positive populations. This study assessed the seroprevalence, epidemiology and laboratory characteristics of these two parasitic infections in a non-endemic setting in an immigrant/refugee HIV positive community.

**Methods:**

Between February 2015 and 2018 individuals born outside of Canada receiving care at the centralized HIV clinic serving southern Alberta, Canada were screened by serology and direct stool analysis for schistosomiasis and strongyloidiasis. Canadian born persons with travel-based exposure risk factors were also screened. Epidemiologic and laboratory values were analyzed using bivariate logistic regression. We assessed the screening utility of serology, direct stool analysis, eosinophilia and hematuria.

**Results:**

253 HIV positive participants were screened. The prevalence of positive serology for *Schistosoma* and *Strongyloides* was 19.9 and 4.4%, respectively. Age between 40 and 50 years (OR 2.50, 95% CI 1.13–5.50), refugee status (3.55, 1.72–7.33), country of origin within Africa (6.15, 2.44–18.60), eosinophilia (3.56, 1.25–10.16) and CD4 count < 200 cells/mm^3^ (2.46, 1.02–5.92) were associated with positive *Schistosoma* serology. Eosinophilia (11.31, 2.03–58.94) was associated with positive *Strongyloides* serology. No *Schistosoma* or *Strongyloides* parasites were identified by direct stool microscopy. Eosinophilia had poor sensitivity for identification of positive serology. Hematuria was not associated with positive *Schistosoma* serology.

**Conclusion:**

Positive *Schistosoma* and *Strongyloides* serology was common in this migrant HIV positive population receiving HIV care in Southern Alberta. This supports the value of routine parasitic screening as part of standard HIV care in non-endemic areas. Given the high morbidity and mortality in this relatively immunosuppressed population, especially for *Strongyloides* infection, screening should include both serologic and direct parasitological tests. Eosinophilia and hematuria should not be used for *Schistosoma* and *Strongyloides* serologic screening in HIV positive migrants in non-endemic settings.

## Background

Schistosomiasis and strongyloidiasis are helminth infections endemic to parts of Africa, Asia and Latin America. Unlike most protozoa, S. *stercoralis* (St) and *Schistosoma* spp. (Sc) may cause persistent infections lasting decades and without adequate treatment are considered life-long infections [1–3]. Immune suppression, including hematologic malignancy, organ transplantation, Human T-cell Lymphotrophic virus-1 infection and initiation of anti-retroviral therapy (ART) are risk factors for St hyperinfection syndrome, which is characterized by high parasite burden, and disseminated disease [4]. In people living with Human Immunodeficiency Virus (HIV) and Acquired Immunodeficiency Syndrome (PLWHA), Immune Reconstitution Inflammatory Syndrome (IRIS), the use of corticosteroids to control IRIS [5–14], and the increase in the invasive S. *stercoralis* filariform larvae with ART initiation likely all play a role [7, 15] in the increased risk of St hyperinfection and disseminated disease. Disseminated Sc infections occurring with ART initiation have also been described and in some cases attributed to IRIS [16, 17]. Treatment of helminth infections in PLWHA decreases rates of HIV transmission mainly by decreasing the HIV viral load (VL) [18–22]. Helminth treatment also has a positive impact on untreated HIV infection; decreases in HIV VL are observed when persons are dewormed and treatment of schistosomiasis slows the decline in CD4 count [20–22].

Screening recommendations for parasitic infection in refugees and immigrants vary widely. Canadian guidelines recommend serologic screening for all refugees from Africa for schistosomiasis and refugees from Africa, Asia and South East Asia for strongyloidiasis [23]. European guidelines endorse a similar serological screening approach [24]. Guidelines in the United States (US) support presumptive treatment with praziquantel for schistosomiasis for refugees from Africa. For refugees from Africa, Asia, Latin America, and the Middle East the US guidelines suggest presumptive treatment of strongyloidiasis with ivermectin [25]. Individuals from *Loa loa* endemic areas should first have infection with *Loa loa* excluded as ivermectin in these individuals can cause life threatening encephalopathy [25]. A Canadian strongyloidiasis advisory group takes a more encompassing screening approach and recommends serologic screening for individuals with epidemiologic risk factors, especially if they have co-morbidities which place them at risk for disseminated disease, such as immunosuppression [26]. This broader approach is endorsed by the Australasian and other international guidelines for migrant populations [27, 28]. Screening for chronic parasitic infections is not addressed in US and World Health Organization HIV care guidelines and European guidelines only briefly mention Sc serology depending on travel or place of origin [29–31].

There is limited information on screening for Sc and St infections in non-endemic settings in PLWHA. The acute and chronic complications attributable to HIV-helminth co-infection are thought to be under recognized [5]. Serious clinical outcomes arise when the diagnosis of disseminated helminth infection is delayed. Support for early diagnosis of HIV-helminth co-infection in PLWHA allows for appropriate and timely treatment. We hypothesized Sc and St helminth infections occur at significantly high rates in our cohort of migrant PLWHA to warrant screening as part of standard HIV care. To investigate this question, we screened for Sc and St infection in all non-Canadian born patients receiving HIV care in Southern Alberta over a three-year period. We measured the seroprevalence of helminth infection and identified epidemiologic and laboratory variables to inform more focused future helminth screening in this migrant population receiving HIV care in Southern Alberta. Finally, serology, direct stool analysis, eosinophilia and hematuria were evaluated for their utility as screening tests for helminth seropositivity in migrant PLWHA.

## Methods

### Population & Study Design

This cross-sectional study enrolled PLWHA receiving care at the Southern Alberta Clinic (SAC) in Calgary. This clinic provides centralized HIV care, including pharmacy and social work services, to all PLWHA in Southern Alberta, Canada. Approximately 50% of the clinic’s patient population are immigrants. Between February 2015 and February 2018 serologic screening for Sc and St was performed for all new (at clinic intake as part of baseline bloodwork) and current (included as part of scheduled bloodwork) SAC patients born outside of Canada or with travel risk factors for exposure. A three-year screening period was chosen to balance an adequate period for data collection with time to analysis to ascertain whether the data supported the allocation of resources for continued screening. Testing was performed on all immigrant clinic patients as part of routine care. The data analysis was undertaken as part of a quality improvement initiative as defined by our Bioethics committee.

### Parasitic testing

Screening in this non-endemic setting was performed with stool ova and parasitic microscopic examination and serology. Participants were given a take home stool specimen collection kit after receiving instructions on collection. For each stool sample collected a single direct smear was examined using the Kato-katz (Kk) technique for a minimum of 15 min. Direct smear was used over other direct parasitological tests as Kk is the only locally available test for screening. The Sc and St enzyme immunoassay (EIA) IgG serology testing was performed at the National Reference Centre for Parasitology in Montreal, Canada [32] with results reported as negative, intermediate (non-negative but not reaching the laboratory defined cut-off value for a clearly positive result) or positive. The Sc EIA uses a pooled extract of antigens from adult S*. mansoni* and S. *haematobium* and has a sensitivity of approximately 90% [32]. An optical density ≥ 0.5 is indicative of infection at some unknown time and the test detection capabilities for S*. japonicum* and S*. mekongi* are not well established [32]. The St EIA uses a recombinant antigen developed by the National Institutes of Health known as NIE and has a sensitivity of 85% [32]. We considered non-negative serology results as an estimate of prevalence as no stool samples were positive for Sc or St. Serology was chosen as other tests, such as point of care (POC) antigen based testing and molecular tests, were not available in our Canadian non-endemic setting for clinical testing. All participants with positive/intermediate serology results were reviewed by a tropical medicine specialist for consideration of appropriate treatment.

### Epidemiologic & Laboratory variables

All epidemiologic data was collected during the patient’s first clinic visit through a one-on-one interview with a clinic nurse, aided if necessary, by a third-party interpreter. For participants already receiving care at the clinic epidemiologic data from their first clinic visit was utilized. Most participants were unable to recollect whether they had received previous parasitic treatment. Countries of origin were grouped according to United Nation geographic regions. All epidemiologic variables collected are listed in Table 1. A complete blood count (CBC) and urinalysis were included in the analysis if performed within three months prior to serology. Macroscopic examination and dipstick testing were performed on all urines and additional microscopic analysis was performed if a sample had abnormalities including the presence of blood. Stool samples collected up to one-month post-serology were included, thereby accommodating for any required repeat stool collection. Analyses for association of positive/intermediate serology with serum eosinophilia and hematuria were performed including all participants. The analyses were repeated excluding participants with evidence of several different parasitic infections, however these analyses were not included as the results were unchanged. Associations with eosinophilia were evaluated using two cut offs; ≥ 0.5x10^9^cells/L, common in parasitic literature, and > 0.7x10^9^cells/L, used in our local setting to define eosinophilia.

**Table 1:** Epidemiologic characteristics and association with positive serology

Characteristic	Number (%)	Positive^†^ *Schistosoma* serology			Positive^†^ *Strongyloides* serology		
		Number (%)	OR (95% CI)	*p*-value	Number (%)	OR (95% CI)	*p*-value
Sex							
Female	88 (34.78)	17 (19.32)	1.00 (referent)		4 (4.54)	1.10 (.23–4.48)	1.00^#^
Male	164 (64.82)	30 (18.29)	.96 (.49–1.85)	.890	7 (4.27)	.91 (.22–4.36)	1.00^#^
Age							
< 30	40 (15.81)	5 (12.50)	0.97 (.31–2.98)	0.961	0 (0.00)	–	–
30- < 40	94 (37.15)	12 (12.77)	1.00 (referent)	–	7 (7.45)	3.08 (.76–14.76)	0.13^#^
40- < 50	80 (31.62)	21 (26.25)	**2.50 (1.13–5.50)**	**0.023**	1 (1.25)	.21 (.0048–1.52)	0.18^#^
> 50	39 (15.42)	9 (23.08)	2.31 (.87–6.12)	0.092	3 (7.69)	2.17 (.35–9.60)	0.45^#^
Diagnosis HIV							
New	77 (30.56)	11 (14.29)	.61 (.39–1.27)	0.185	1 (1.30)	.22 (.0049–1.58)	0.20^#^
Established	175 (69.44)	36 (20.57)	1.00 (referent)	–	10 (5.71)	4.61 (.64–203.55)	0.20^#^
ART treatment							
Active ART	134 (52.96)	20 (14.93)	1.00 (referent)		6 (4.48)	1.00 (referent)	–
No current ART	119 (47.04)	27 (22.69)	1.55 (.82–2.95)	0.183	5 (4.20)	0.92 (.27–3.10)	0.900
Immigration status							
Citizen	69 (28.75)	11 (15.94)	0.74 (0.32–1.64)	0.56^#^	1 (1.45)	.30 (.0067–2.28)	0.416^#^
Permanent Resident	134 (55.83)	30 (22.39)	1.70 (0.82–3.66)	0.169^#^	6 (4.48)	1.63 (0.34–10.29)	0.743^#^
Temporary resident	37 (15.42)	4 (10.81)	0.52 (0.13–1.62)	0.354^#^	2 (5.41)	1.60 (0.156–8.902)	0.831^#^
Refugee status^*^							
Non refugee	206 (81.42)	30 (14.56)	1.00 (referent)	–	8 (3.89)	0.59 (0.13–3.59)	0.66^#^
Refugee	47 (18.58)	17 (36.17)	**3.55 (1.72–7.33)**	**0.001**	3 (6.38)	1.70 (0.28–7.45)	0.66^#^
Region of origin							
Africa	145 (57.54)	40 (27.59%)	**6.15 (2.44–18.60)**	**0.000**^#^	9 (6.21)	3.50 (0.704–34.01	0.16^#^
Latin America	36 (14.29)	2 (5.56)	0.25 (.028–1.06)	0.065^#^	1 (2.78)	0.60 (.013–4.45	1.00^#^
North America	10 (3.97)	2 (20.00)	1.03 (.103–5.41)	1.000^#^	0 (0.00)	–	
Other	4 (1.59)	0 (0.00)	–	–	1 (25.00)	7.64 (.14–105.60)	0.33^#^
Asia	57 (22.62)	2 (3.51)	**0.12 (.14–.51)**	**0.0006**^#^	0 (0.00)	–	–

N total = 253. The number of observations are listed for each category with percentage in brackets. ^**†**^Positive serology refers to all non-negative serology results. ^#^Exact logistic regression was used for analyses with cell counts less than 5. Odds ratios (ORs) are listed for each comparison with 95% confidence intervals (CI) in brackets. Bolded CIs and *p*-values are statistically significant. *Refugees included those that were Government assisted, privately sponsored, refugee claimants and refugees otherwise unspecified.

### Statistical analysis

Statistical analysis was performed using STATA version 12.0. Variables were analyzed using bivariate logistic regression. Exact logistic regression was used for analyses that included cell counts with fewer than five observations. Regression results were reported as odds ratios (ORs) with an associated 95% confidence interval (CI) for participants with positive/intermediate Sc serology and separately for participants with positive/intermediate St serology. *P*-values < 0.05 were considered significant.

## Results

The study enrolled 253 participants and 185 (73.1%) were screened for chronic parasitic infection within one month of clinic enrollment. 64 (25.3%) participants had their screening performed between one and three months after clinic enrollment and 4 (1.6%) were screened after more than three months. At the time of serology 30.6% of participants had a new diagnosis of HIV and 53.0% were on ART. Refugees accounted for 18.6% of participants. The most common region of origin was Africa (57.5%) and specifically East Africa (91 participants, 36.1%). The second most common region of origin was Asia of which 34 participants (13.5%) were from South East Asia. Epidemiologic characteristics are shown in Table 1.

### Parasitic screening

Most study participants had screening serology results for schistosomiasis (236, 93.3%) and strongyloidiasis (249, 98.4%). 47 participants (19.9%) had a positive (38, 80.9%) or intermediate (9, 19.1%) result for Sc serology while 11 participants (4.4%) had a positive (9, 81.8%) or intermediate (2, 18.2%) result for St serology. Two participants had positive serology for both parasites. No stool samples were positive on direct stool microscopy for either Sc or St, therefore prevalence estimates were solely based on serology.

### Epidemiologic indices

Results of the bivariate analysis are shown for the individual serologic results in Table 1. Participant sex and having a new diagnosis of HIV had no association with serology. Participants between 40 and 50 years of age (OR 2.5, 1.13–5.50) were more likely to have positive Sc serology. Although not statistically significant, persons not currently on ART (OR 1.55, 0.82–2.95) showed a trend towards increased odds of having positive Sc serology.

A region of origin in Africa (OR 6.15, 2.44–18.60) significantly increased the odds of having positive Sc serology. No statistically significant region of origin association was identified for St serology, although 81.8% of participants with positive St serology were from Africa. Refugees were more likely to have positive Sc serology (OR 3.55, 1.72–7.33) although notably a significant proportion of non-refugee immigrants also had positive Sc serology (30, 14.6%). Refugee status was not associated with positive St serology and immigration status had no association with serology.

### Laboratory indices

CD4 count < 200 cells/mm^3^ was associated with positive Sc serology (OR 2.46, 1.02–5.92). Participants with a detectable VL (OR 1.59, 0.83–3.07) showed a trend towards increased odds of having positive Sc serology (Table 2). Only 148 participants (58.5%) submitted stool samples allowing for paired analysis of direct ova and parasite microscopy with serology. Even when specimens were serially submitted, one fifth (20.8%) of the samples were unacceptable for analysis because of container overflow. Using the Kk technique, no stool samples were positive for Sc or St; however, one case of giardia and one case of hookworm were identified. Non-pathogenic parasites were seen in 12/117 (10.3%) of participants including B*. hominis* (6, 5.1%) and D*. fragilis* (2, 1.7%).

**Table 2:** Laboratory characteristics and association with positive serology

Characteristic	Number (%)	Positive^†^ *Schistosoma* serology			Positive^†^ *Strongyloides* serology		
		Number (%)	OR (95% CI)	*p*-value	Number (%)	OR (95% CI)	*p*-value
CD4 count cells/mm^3^							
< 200	53 (21.12)	15 (28.30)	**2.46 (1.02–5.92)**	**0.046**	3 (5.66)	1.60 (0.26–7.32)	0.731^#^
200–350	55 (21.91)	11 (20.00)	1.8 (.71–4.57)	0.217	3 (5.46)	1.56 (.25–7.14)	0.757^#^
350–500	65 (25.90)	10 (15.39)	1.06 (.42–2.69)	0.901	1 (1.54)	0.32 (.0070–2.35)	0.45^#^
> 500	78 (31.08)	11 (14.10)	1.00 (referent)	–	3 (3.85)	.94 (.15–4.28)	1.00^#^
Viral load							
Suppressed	123 (48.62)	18 (14.63)	1.00 (referent)	–	5 (4.07)	1.00 (referent)	–
Not suppressed	130 (51.38)	29 (22.31)	1.59 (0.83–3.07)	0.162	6 (4.62)	1.12 (0.33–3.79)	0.853
Stool analysis							
Negative for parasites	103 (88.03)	17 (16.51)	0.54 (.14–2.65)	0.52^#^	5 (4.85)	0.68 (.068–34.46)	1.00^#^
Positive schisto/strongy	0 (0.00)	0 (0.00)	–	–	0 (0.00)	–	–
Positive for other parasites*	14 (11.97)	4 (28.57)	1.85 (.38–7.43)	0.52^#^	1 (7.14)	1.47 (.029–14.75)	1.00^#^
Urinalysis							
Hematuria	31 (13.42)	8 (25.81)	1.48 (.61–3.60)	0.382	N/A	N/A	N/A
No hematuria	200 (86.58)	37 (18.50)	1.00 (referent)	–	N/A	N/A	N/A
Eosinophilia > 0.7x109cells/L							
No eosinophilia	226 (92.24)	38 (16.81)	1.00 (referent)	–	5 (2.21)	**.089 (.017–.49)**	**0.0055**^#^
Eosinophilia	19 (7.76)	7 (36.84)	**3.56 (1.25–10.16)**	**0.018**	4 (21.05)	**11.31 (2.03–58.94)**	**0.0055**^#^
Eosinophilia ≥0.5x10^9^cells/L							
No eosinophilia	214 (84.58)	36 (16.82)	1.00 (referent)		4 (1.87)	**0.10 (.019–0.51)**	**0.0045**^#^
Eosinophilia	31 (12.65)	9 (29.03)	**2.44 (1.01–5.91)**	**0.048**	5 (16.13)	**9.73 (1.96–52.33)**	**0.0045**^#^

N total = 253. The number of observations are listed for each category with percentage in brackets. ^**†**^Positive refers to all non-negative serology results. ^#^Exact logistic regression was used for analyses with cell counts less than 5. Odds ratios (ORs) are listed for each comparison with 95% confidence intervals (CI) in brackets. Bolded CIs and *p*-values are statistically significant. *Other parasites included B. *hominis D. fragilis.*

Serology could be paired with urinalysis (91.3%) and CBC (96.8%) for most participants. 31 participants (13.4%) had hematuria on urinalysis although the presence of hematuria was not associated with positive Sc serology (Table 2). 19 participants (7.8%) had eosinophilia based on a cut-off of > 0.7x10^9^cells/L and 31 (12.7%) using a less stringent cut-off of ≥0.5x10^9^cells/L (Table 2). Eosinophilia, regardless of cut-off, was positively associated with both positive Sc (OR 3.56, 1.25–10.16) and St serology (OR 11.31, 2.03–58.94). Eosinophilia using a cut-off of > 0.7x10^9^cells/L as a screening test for chronic parasitic infection had low sensitivity at 15.6% (20.0%, cut off ≥0.5x10^9^cells/L) in identifying participants with positive Sc serology and 44.4% (55.6%, cut off ≥0.5x10^9^cells/L) for positive St serology. The negative predictive value was 82.1% (82.2%, cut-off ≥0.5x10^9^cells/L) and 97.8% (98.1%, cut-off ≥0.5x10^9^cells/L) for Sc and St serology, respectively. The specificity of eosinophilia was high at 95.1% (90.7%, cut-off ≥0.5x10^9^cells/L) for positive Sc serology and 93.5% (88.8%, cut-off ≥0.5x10^9^cells/L) for positive St serology.

## Discussion

Schistosomiasis and strongyloidiasis seroprevalence in migrants worldwide range from 1.4 to 73.0% and 1.9 to 31.4%, respectively [33]. These wide ranges reflect global variation in migrants’ regions of origin and changes in migration patterns over time. They also reinforce the need for contemporary region and population specific epidemiologic data to inform health care practices.

Refugee status is a recognized risk factor for chronic parasitic infection [34, 35]. However, we also identified positive Sc serology in a significant number of non-refugee immigrants (14.6%). Worldwide, immigrants represent a much larger population than refugees, although they often receive less health screening compared to refugees [36]. Indeed, many immigrants have similar exposure risk factors for parasitic infection, but are not captured in most screening guidelines and practices in Canada and worldwide [23, 25]. Although our study focused on PLWHA, a broader implication is that a significant number of chronic parasitic infections remain undetected in immigrant populations. Therefore, screening should be extended to immigrant populations with exposure risk factors regardless of their HIV status.

This study found that participants with markers of poor HIV control, specifically low CD4 count, a detectable VL and no current ART were more likely to have positive Sc serology. In contrast, Hochberg et al. found no association with CD4 count although their data were collected nearly ten years ago and used a distinct serologic test in a population with more poorly controlled HIV [37]. Poor HIV control, which often co-exists with poor access to health care, may be a risk factor for chronic parasitic infection. Our results reinforce the vulnerability of certain HIV positive migrants and underscore the importance of providing health care tailored to their needs, which may include parasitic screening.

Challenges exist with the serologic assays used in this study. Sc serology cannot distinguish between current and previously treated infections and the Sc assay used in this study cannot be correlated with worm or egg burden [32]. In contrast St serologic titres using the NIE recombinant antigen decline by more than 50% in 81.2% of patients following adequate treatment. Repeat titres are negative in 72.5% of patients following adequate treatment [38]. The false positive rate for the St serology assay is approximately 5% in persons known to be infected with filaria, Sc, or *Echinoccocus* [32]. Likewise, there may be cross-reactions with the Sc EIA for persons infected with filaria, St, *Echinococcus* or T*. solium*, which would overestimate seroprevalence. Few stool positive samples have been tested as part of validation of the Sc serology assay, therefore test sensitivity and specificity are estimates [32]. The Sc and St EIA assays have a sensitivity of approximately 90 and 85%, respectively and there is limited data on the performance of these assays, and serology assays more generally, in PLWHA. Luvira et al. found the sensitivity of a distinct St serologic assay was decreased in a range of immunocompromised (42.9%) as compared to immunocompetent (96.0%) hosts [39]. In our analysis, we based seroprevalence estimates on combined positive and intermediate serology results because the serologic assays likely underestimate true prevalence, especially in an immunocompromised population. If appropriate, it is our practice to offer treatment to all PLWHA with non-negative serology as they comprise a high-risk group for complications of helminth infection and treatments are generally well tolerated. Screening for St and Sc in our setting would be well suited to integrated serosurveillance: testing platforms that combine multiplex serologic assays against multiple pathogens. Although not yet routinely available, integrated serosurveillance would increase the testing value and efficiency of this type of work [40].

In our study, stool collection rates were poor (58.4%) compared to serology (> 93%) and samples were often unsuitable for analysis because of container overflow. Communication barriers in explaining correct stool sample collection and the requirement for patients to drop off samples at a later date likely contributed to stool data incompleteness. This reflects a real world outpatient screening experience. In this non-endemic setting the only direct parasitological test routinely available for clinical screening is the Kk direct smear technique. No Sc or St ova or parasites were identified in 117 samples analyzed, likely due to the low sensitivity of a single direct smear. Although repeated stool sampling likely increases the sensitivity of the Kk technique, our outpatients report an unwillingness to submit serial samples because of perceived inconvenience. Other techniques, such as the Baerman technique or the stool agar culture method, have higher sensitivity [41] but are more labour intensive and pose a greater biohazard risk to laboratory technicians. The lower likelihood of a positive direct stool examination in light intensity infections [42, 43], a common occurrence in the context of screening, likely also contributed to poor stool detection rates. POC circulating cathodic antigen testing for Sc [44] and nucleic acid based diagnostic techniques have higher sensitivity than direct smear analysis [45, 46] but currently are not routinely available for clinical screening in our non-endemic setting. Although emerging technologies are promising, a persistent difficulty in the diagnosis of helminth infections is the lack of a gold standard [47]. Despite its limitations, direct stool examination should have a role in screening in non-endemic settings when other direct parasitological tests or newer more sensitive tests are not routinely available. Our suggestion places high value in recognizing that serologic assays may have lower sensitivity in immunocompromised hosts and the high degree of morbidity and mortality, especially with St infections, in PLWHA. This endorsement is in concordance with the recommendation of a recent guideline for screening of strongyloidiasis in immunosuppressed people in non-endemic countries [27].

Eosinophilia was associated with both positive Sc and St serology. A limitation in the interpretation of this association is that other causes of eosinophilia, such as malignancy and fungal infection [48], were not investigated. Laboratory data were collected at a single time point; therefore, assumptions cannot be made regarding causality between laboratory data, including eosinophilia, and positive serology. Other researchers have found that eosinophilia is less pronounced in immunosuppressed persons with strongyloidiasis, including those with HIV, chronic illness and taking immunosuppressants as compared to otherwise healthy subjects [49]. Using a lower cut-off of > 0.4x10^9^cells/L to define eosinophilia, Hochberg et al. found eosinophilia was associated only with positive Sc serology in a cohort of patients with poorly controlled HIV [37]. Our work suggests that within a subpopulation of persons who are relatively immunosuppressed by HIV, eosinophilia is a useful indicator of positive Sc and St serology as a marker of parasitic infection. It is unclear whether this remains the case in advanced HIV as our study had very few participants (15, 5.9%) with a CD4 count < 50 cells/mm^3^. Given that not all persons with positive serology have eosinophilia, a CBC is not an appropriate screening test to replace serologic testing. In this study eosinophil counts ≤0.7x10^9^cells/L were seen in 84.4 and 55.6% of participants with positive Sc and St serology, respectively. Eosinophilia had high specificity for positive Sc and St serology with improved test performance using a higher cut-off for the definition of eosinophilia. Therefore, the presence of eosinophilia should be further investigated. Investigations should include a detailed travel history and both serology and serial stool microscopy to look for the presence of a parasitic infection, including non-Sc and St infections. If the etiology of eosinophilia remains in question, techniques with higher sensitivity, such as the Baerman method, should be employed.

Hematuria was not associated with positive Sc serology. It is well established that urogenital schistosomiasis, caused by S*. haematobium*, often causes hematuria [50]. Our serologic assay used an antigen extract of S*. haematobium* and S*. mansoni* and, thus infections with S*. mansoni* may have diluted the association between hematuria and Sc serology.

This study had a relatively small sample size, in part because participants comprised a highly selected population of immigrant HIV positive individuals actively seeking HIV care. This negated the ability to perform a multivariate regression analysis to account for dependent variables. Sample size also precluded identification of any epidemiologic factors to guide screening for positive St serology as a marker of strongyloidiasis. Currently, there is no data from HIV positive or immunosuppressed cohorts estimating rates of complications, morbidity and associated health care costs attributable to chronic parasitic infection. Our knowledge of predisposing factors to development of severe forms of strongyloidiasis are based solely on case reports [8, 9, 11, 12] and case series [51]. Given the risks associated with strongyloidiasis, namely hyperinfection and disseminated disease, it is prudent to continue screening PLWHA from the tropics and subtropics as part of HIV care until more data is available to tailor a screening approach. Due to very small numbers locally we did not include undocumented HIV migrants to Canada.

Areas that warrant further research include serologic test performance in migrant PLWHA living in non-endemic settings and the degree of eosinophilia in relation to markers of HIV control, including CD4 count and VL. The etiology of the high degree of hematuria seen in this study (13.4%) undoubtedly has multiple causes and warrants further exploration. Surveying attitudes and current practices regarding chronic parasitic infection screening at other HIV treatment centres across the country and internationally will be important in identifying potential areas where patient care can be improved.

## Conclusions

The Sc and St seroprevalence in this cohort of mainly foreign-born PLWHA receiving HIV care in Southern Alberta, Canada was 19.9 and 4.4%, respectively. Given the significant morbidity and mortality associated with helminth infections in PLWHA we support Sc and St screening as a routine component of HIV care in non-endemic settings. Screening should be performed with serology and a direct parasitological test. Screening efforts for Sc should be focused on specific groups of people: those originating from or migrating through Africa; refugees; people aged 40 to 50 years; individuals with eosinophilia; or individuals with markers of poorly controlled HIV (i.e. CD4 counts < 200 cells/mm^3^, a detectable VL or no current ART). The low St seroprevalence precluded more specific recommendations on focused screening efforts for strongyloidiasis. Eosinophilia and hematuria should not be used as a screening test for positive Sc and St serology in PLWHA in non-endemic settings.

## Data Availability

The dataset used during the current study is available from the corresponding author on reasonable request.

## Abbreviations

ART: Anti-retroviral therapy
CBC: Complete blood count
CI: Confidence interval
EIA: Enzyme immunoassay
HIV: Human Immunodeficiency Virus
IRIS: Immune Reconstitution Inflammatory Syndrome
Kk: Kato-katz
OR: Odds ratio
PLWHA: People living with HIV and Acquired Immunodeficiency Syndrome
POC: Point of care
SAC: Southern Alberta Clinic
Sc: *Schistosoma* spp.
St: S. *stercoralis*
US: United States
VL: Viral load
